# Treatment Effect of Beta-Blocker Therapy After Acute Myocardial Infarction With Preserved or Mildly Reduced Ejection Fraction: A Systematic Review and Trial Sequential Analysis

**DOI:** 10.7759/cureus.111154

**Published:** 2026-06-19

**Authors:** Christopher Chinnatambi, Louise Sakowski, Abdul-Rahaman Adedolapo Ottun, Prince K Darko, Baffour Otchere, Brice Njobe, Ohene Poku Agyemang, Jorge Pimentel, Wael Kanjo, Sammudeen Ibrahim

**Affiliations:** 1 Internal Medicine, Piedmont Athens Regional Medical Center, Athens, USA; 2 Cardiology, Piedmont Macon Medical Center, Macon, USA; 3 Graduate Medical Education, Piedmont Athens Regional Medical Center, Athens, USA; 4 Internal Medicine, Ryazan State Medical University, Ryazan, RUS; 5 Cardiology, Piedmont Athens Regional Medical Center, Athens, USA

**Keywords:** acute myocardial infarction, beta-blockers, heart failure, major adverse cardiovascular events, preserved ejection fraction, secondary prevention

## Abstract

Long-term use of beta-blockers has been the standard of care after acute myocardial infarction (AMI), but evidence comes largely from pre-reperfusion-era trials in patients with reduced left ventricular ejection fraction (LVEF). With modern reperfusion and optimal secondary prevention, the benefit in patients with preserved and mildly reduced LVEF remains uncertain. Most guidelines continue to recommend routine beta-blocker use in this population, yet no prior meta-analysis has been restricted to recent randomized controlled trials (RCTs).

The objective of this article was to evaluate the efficacy and safety of beta-blocker versus no beta-blocker therapy after AMI in patients with preserved or mildly reduced LVEF.

The Cochrane Central, PubMed, Embase, and ClinicalTrials.gov were searched for RCTs that compared beta-blocker versus no beta-blocker therapy after AMI in patients with preserved LVEF (≥50%) or mildly reduced LVEF (40-49%). The primary outcome was major adverse cardiovascular events (MACE); secondary outcomes included all‑cause mortality, cardiac death, myocardial infarction (MI), unplanned revascularization, heart failure (HF) events, malignant ventricular arrhythmia, and stroke. Trial sequential analysis (TSA) was performed to assess the conclusiveness of the evidence and whether further trials are warranted.

Four RCTs comprising 19,826 patients (BB (beta blocker), n = 9892; no BB, n = 9934) were included. Beta-blockers did not significantly reduce MACE (RR 0.95, 95% CI 0.87-1.03). No difference was observed in all-cause mortality (RR 0.98, 95% CI 0.86-1.13), cardiac death (RR 1.15, 95% CI 0.88-1.51), MI (RR 0.88, 95% CI 0.75-1.04), unplanned revascularization (RR 1.01, 95% CI 0.88-1.16), HF events (RR 0.83, 95% CI 0.64-1.08), malignant ventricular arrhythmia (RR 0.84, 95% CI 0.47-1.50), or stroke (RR 1.18, 95% CI 0.85-1.64). Heterogeneity was low across outcomes. TSA demonstrated that the cumulative evidence for all-cause mortality, unplanned revascularization, and malignant ventricular arrhythmia was conclusive, while MACE and other outcomes remain inconclusive.

In AMI patients with preserved or mildly reduced LVEF, beta-blockers did not reduce ischemic events, mortality, or arrhythmia. TSA suggests that additional trials are unlikely to alter conclusions for mortality and revascularization, but uncertainty persists for composite outcomes.

## Introduction and background

Beta-adrenergic blockers have been a cornerstone of secondary prevention after acute myocardial infarction (AMI) for more than four decades. Landmark trials conducted in the 1980s demonstrated substantial reductions in reinfarction, arrhythmic death, and all-cause mortality with beta-blocker therapy in post-myocardial infarction (MI) patients [1-6]. These benefits led to the widespread adoption of long-term beta-blocker therapy after MI. Accordingly, current American College of Cardiology/American Heart Association guidelines provide a Class I recommendation for beta-blocker therapy in AMI survivors with left ventricular ejection fraction (LVEF) >40%, while the European Society of Cardiology assigns a Class IIa recommendation [7,8]. However, these early trials were conducted in the pre-reperfusion era, before the routine use of primary percutaneous coronary intervention (PCI), dual antiplatelet therapy (DAPT), high-intensity statins, and angiotensin-converting enzyme inhibitors (ACEIs) [9]. With advances in reperfusion and pharmacotherapy, the absolute risk of arrhythmic and ischemic complications in modern post-MI patients has fallen substantially; therefore, raising the question of whether the legacy evidence remains applicable today [10].

While the benefit of beta-blockers remains well-established in patients with reduced LVEF (<40%), their role in patients with preserved (≥50%) or mildly reduced (40-49%) LVEF is less clear [11,12]. In the contemporary era, observational studies and registries have challenged the universal benefit of beta-blockers in this population. For example, the Reduction of Atherothrombosis for Continued Health (REACH) registry found no significant association between beta-blocker use and improved cardiovascular outcomes among patients with prior MI receiving optimized medical therapy [13]. Similarly, a large meta-analysis by Bangalore et al. reported that beta-blockers reduced events in the pre-reperfusion era but did not confer a significant mortality benefit in the reperfusion era [14].

More recently, several randomized controlled trials (RCTs) have sought to address this evidence gap, with conflicting results [15-17]. Therefore, a comprehensive systematic review and meta-analysis of contemporary RCTs is needed. To our knowledge, this is the first meta-analysis restricted to randomized trials exclusively enrolling patients with preserved or mildly reduced LVEF, directly addressing this important gap in secondary prevention after AMI.

## Review

This systematic review and meta-analysis was conducted following the Preferred Reporting Items for Systematic Reviews and Meta-analyses (PRISMA) methodology and the Cochrane protocol [18,19]. Additionally, the prospective meta-analysis protocol was registered in the International Prospective Register of Systematic Reviews (PROSPERO), with registration number CRD420251142277 [20]. Given that the data used for the analysis are publicly available and de-identified, the Institutional Review Board deemed the study exempt. Upon request, further data supporting this study’s findings are available from the corresponding author.

Three authors (SI, HN, ED) independently queried the Cochrane Central Register of Controlled Trials, PubMed/MEDLINE, EMBASE, Web of Science, and ClinicalTrials.gov databases from 2010 through September 2025 for all RCTs that compared beta-blocker versus no beta-blocker therapy after AMI in patients with preserved or mildly reduced LVEF (≥40%). The following keywords were used for the search: “Beta-blocker,” “Beta adrenergic antagonist,” “Acute myocardial infarction,” “STEMI,” “NSTEMI,” “Post-myocardial infarction,” “Preserved ejection fraction,” and “LVEF >40%”. These authors reviewed the studies independently for eligibility according to the pre-specified eligibility criteria. All discrepancies were resolved via a panel discussion among authors.

We limited our search to RCTs conducted after 2010. Other than that, we imposed no restrictions based on publication status or language. We considered studies eligible if they (1) were RCTs, (2) enrolled patients with AMI within 7-14 days of index MI, (3) included patients with LVEF ≥ 40%, and (4) compared beta-blocker versus no beta-blocker therapy. We included data from published articles regardless of race or ethnicity. Exclusion criteria were studies restricted only to patients with a history of MI or stable coronary artery disease without a recent AMI, or trials that did not report any of our predefined clinical endpoints of interest.

Our primary endpoint was major adverse cardiovascular events (MACE), in most studies defined as a composite of all-cause mortality, MI, unplanned revascularization, heart failure (HF), and stroke. Given the variability in the definition of MACE across included trials, we harmonized outcomes by adopting each study’s reported composite endpoint as defined by the original investigators. While individual components varied (e.g., inclusion of stroke or revascularization), all definitions included core cardiovascular outcomes such as all-cause mortality and MI. We therefore considered these endpoints sufficiently comparable for pooled analysis, consistent with prior meta-analyses of composite cardiovascular outcomes. See detailed definitions per included trials in Table 1. Secondary endpoints included all-cause mortality, cardiac death, MI, unplanned revascularization, HF events, stroke, and malignant ventricular arrhythmia (Table 2).

**Table 1:** Definition of MACE in the included trials MACE, major adverse cardiovascular events; MI, myocardial infarction; HF: heart failure

Trial	Definition of MACE / Primary Composite	Components Included
BETAMI-DANBLOCK [17]	All-cause mortality + MI + unplanned revascularization + ischemic stroke + HF + malignant ventricular arrhythmia	Death, MI, UA revascularization, stroke, HF, arrhythmia
REDUCE-AMI [15]	All-cause death + new non-fatal MI	Death, MI
REBOOT-CNIC [16]	Death + reinfarction + HF hospitalization	Death, MI, HF
CAPITAL-RCT [21]	All-cause death, MI, HF	Death, MI, HF (harmonized)

**Table 2:** Secondary endpoints CV: cardiovascular; MI, myocardial infarction; HF: heart failure; PCI: percutaneous coronary intervention; CABG: coronary artery bypass graft surgery

Endpoint	Definition / Comments	Trials Reporting It
All-cause mortality	Death from any cause	All four trials
Cardiac death (CV death)	Death attributed to cardiovascular cause	All trials but BETAMI-DANBLOCK [17]
Recurrent MI	Myocardial infarction meeting standard criteria for 4^th^ Universal Definition	All trials
Unplanned revascularization	PCI or CABG not pre-specified at baseline	All trials but REDUCE-AMI [15]
HF events	Hospitalization or urgent HF worsening requiring therapy	All trials
Malignant ventricular arrhythmia	Sustained ventricular tachycardia, VF, resuscitated arrest	BETAMI-DANBLOCK [17] and REBOOT- CNIC [16]
Stroke	Ischemic or hemorrhagic stroke	All trials

Relevant data were abstracted to the Endnote Reference Manager (version X9.3.3: Clarivate Analytics). The prespecified data points were abstracted and harmonized independently by two authors (CC, AO). The abstraction was independently verified by a third author (LS). Clinical data included baseline and study characteristics, and data on the endpoints of interest, including MACE, all-cause mortality, cardiac death, MI, unplanned revascularization, HF, stroke, and malignant ventricular arrhythmia.

Risk of bias was assessed independently and in tandem for the studies included in compliance with the Cochrane Collaboration RoB 2.0 tool by two authors (CC, AO) [22]. The certainty of evidence was assessed with the Grading of Recommendations Assessment, Development and Evaluation (GRADE) framework [23]. Disagreements were resolved by consensus amongst authors. Publication bias was evaluated through visual inspection of the comparison-adjusted funnel plots, conducted independently by two authors (CC, AO).

Statistical analyses were conducted using Review Manager (version 5.4.1; Copenhagen: The Nordic Cochrane Center, the Cochrane Collaboration, 2020). Mantel-Haenszel risk ratios (RRs) with 95% confidence intervals (CIs) were calculated for all clinical outcomes of interest. The degree of heterogeneity between studies was assessed using both Cochran's Q test and Higgins and Thompsons I2 statistics [24]. For outcomes demonstrating an I² value greater than 50% or p-value <0.10, indicating at least moderate heterogeneity, we predetermined that a leave-one-out sensitivity analysis would be performed to determine the influence of each study on the overall results [25]. The DerSimonian and Laird random-effects model was used to derive pooled effect estimates and to calculate the between-study variance [26].

We conducted several sensitivity analyses to assess the robustness of our findings. First, all outcomes were reanalyzed using pooled inverse-variance hazard ratios. Second, to address uncertainty in the estimation of between-study variance, we applied the inverse-variance restricted maximum-likelihood (REML) method with Hartung-Knapp-Sidik-Jonkman (HKSJ) adjustment for confidence interval estimation. Third, fixed-effect and random-effects models were interconverted to assess the stability of the results across modeling assumptions. Finally, for outcomes demonstrating at least moderate heterogeneity (I² >50% or p <0.10), we prespecified and performed leave-one-out analyses to evaluate the influence of individual trials [27,28].

We performed prespecified subgroup analyses to evaluate whether the effect of beta-blocker therapy on the primary composite outcome of MACE varied according to age (<75 vs ≥75 years), sex, type of MI at index admission (NSTEMI vs STEMI), revascularization strategy (complete vs incomplete), LVEF (40-49% vs ≥50%), and the presence or absence of hypertension, where data was available. We applied Bonferroni correction to subgroup analyses (adjusted α = 0.05/number of hypotheses tested) [29]. In accordance with recent methodological guidance, a p-value for interaction <0.10 was considered evidence of statistically significant heterogeneity of treatment effect between subgroups [30,31].

To address the risks of random error and repeated significance testing in cumulative meta-analysis, we performed trial sequential analysis (TSA) for all outcomes. TSA applies sequential monitoring boundaries, analogous to interim analyses in RCTs, to evaluate whether the accrued evidence is sufficient to confirm or reject a treatment effect. Analyses were conducted using standard software (TSA v0.9.5.10 Beta, Copenhagen, Denmark) with diversity-adjusted required information size (DARIS) estimates calculated from the observed between-trial heterogeneity (D²) [32,33]. We assumed a 10% relative risk reduction (RRR), chosen to represent the smallest clinically meaningful effect, with a two-sided α of 0.05 and power of 80% (1−β). While this threshold was selected to reflect a clinically meaningful effect, smaller treatment effects may still be clinically relevant in this population. Cumulative Z-curves were compared against conventional thresholds and trial sequential monitoring boundaries for benefit, harm, or futility. Crossing the significance boundary indicated firm evidence of benefit or harm, while crossing the futility boundary suggested that additional trials are unlikely to demonstrate the prespecified effect. Boundaries not crossed were considered inconclusive. Exploratory TSA was also performed for the subgroup of patients with LVEF 40-49% vs LVEF ≥50% using the same parameters.

**Figure 1:**
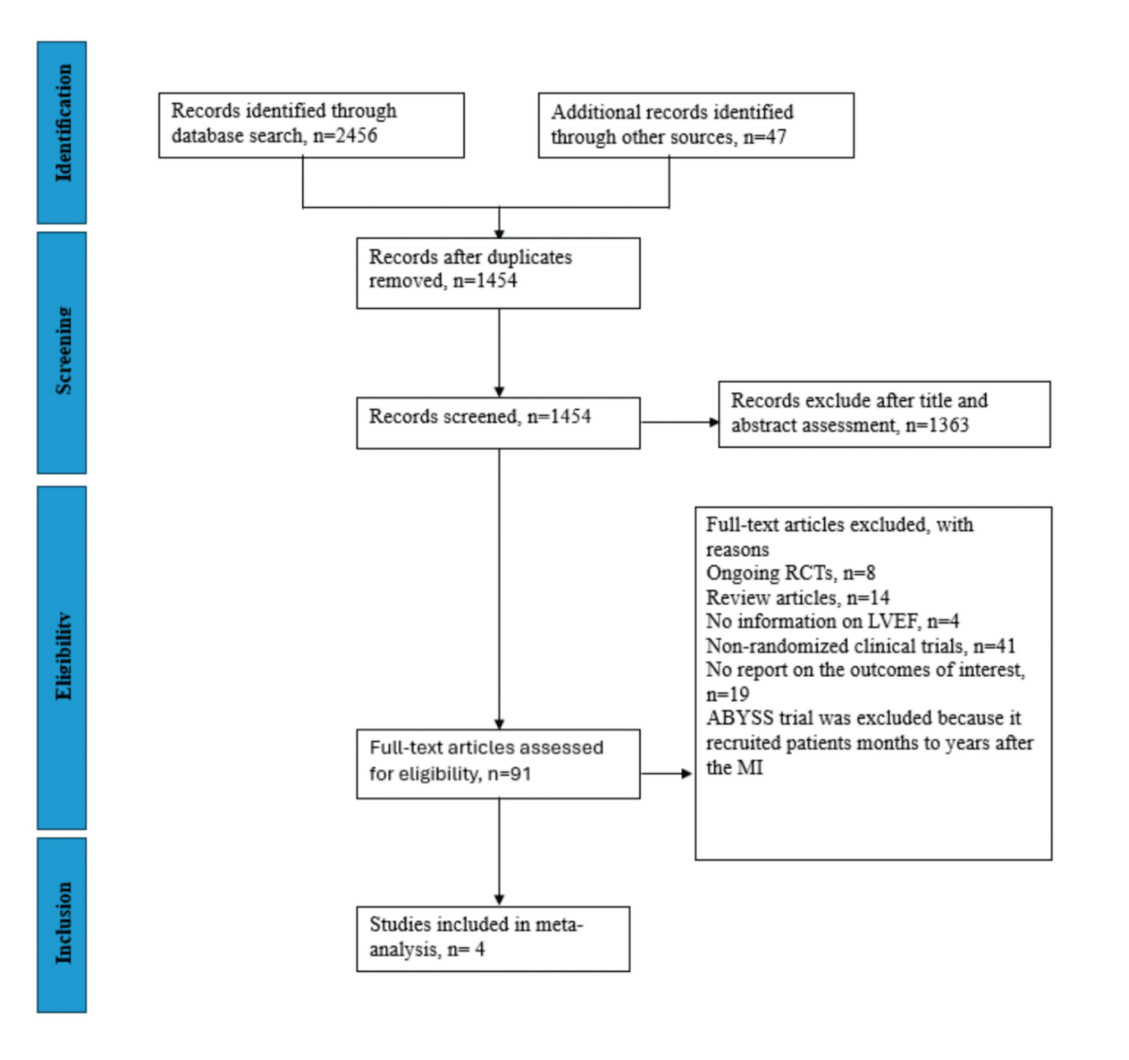
PRISMA flow diagram of study screening and selection PRISMA: Preferred Reporting Items for Systematic Reviews and Meta-Analysis

Our systematic search identified 2503 records from electronic databases and trial registries, of which 91 underwent full-text review. Notably, the ABYSS trial was excluded because it evaluated the safety of beta-blocker discontinuation in stable patients several months to years after MI, rather than initiation of therapy in the acute phase, which was the focus of our study [34]. Ultimately, 4 RCTs enrolling 19,826 patients with AMI and preserved LVEF were included in the quantitative synthesis [15-17,35]. The study selection process is summarized in the PRISMA flow diagram (Figure 1). The mean age of participants was 63.3 years, and 20.6% were female. The weighted median follow-up was 3.6 years, corresponding to 35,784 patient-years in the beta-blocker arm and 35,632 in the control arm.

The baseline and study characteristics of the included RCTs are summarized in Table 1 and Table 3, respectively. The overall risk of bias for the trials included was low. Evidence certainty, assessed using the GRADE framework, was rated as high for most outcomes (Table 4). Assessment of publication bias was limited by the small number of included studies (<10), which precludes meaningful interpretation of funnel plots according to Cochrane guidance.

**Table 3:** Characteristics of the included trials STEMI: ST-segment elevation myocardial infarction; NSTEMI: non-STEMI; LVEF: left ventricular ejection fraction; PCI: percutaneous coronary intervention; AV: atrioventricular; HF: heart failure; COPD: chronic obstructive pulmonary disease; MI: myocardial infarction; ACS: acute coronary syndrome; vtach: ventricular tachycardia; vfib: ventricular fibrillation; EF: ejection fraction

Study	BETAMI-DANBLOCK [17]	CAPITAL-RCT [21]	REBOOT-CNIC [16]	REDUCE-AMI [15]
Registration ID	NCT03646357, NCT03778554	NCT01155635	NCT03596385	NCT03278509
Year	2025	2018	2025	2024
Follow-up, years	3.5	3.9	3.7	3.5
Study Design	Prospective, randomized, open-label, blinded end-point evaluation	Prospective multi-center, open-label, randomized controlled trial	Prospective, randomized, open-label, blinded end- point evaluation	Randomized, parallel-group, open-label trial
Region	Norway, Denmark	Japan	Spain, Italy	Sweden, Estonia, New Zealand
Sample Size	5574	794	8438	5020
Inclusion criteria	Adults 18 years or older with acute myocardial infarction (STEMI or NSTEMI), LVEF ≥ 40%, hemodynamically stable after acute events, revascularization (PCI or thrombolysis, BETAMI only)	Adults 18 years or older hospitalized with acute myocardial infarction, LVEF ≥ 40%, successful primary PCI	Adults 18 years or older with acute myocardial infarction, LVEF ≥ 40%, treated with contemporary guideline-directed therapy, invasive management during index admission (coronary angiography with or without PCI)	Adults 18 years or older at the time of signing the informed consent. Days 1-7 after MI, either STEMI or NSTEMI (Fourth Universal Definition of MI, type 1). Coronary angiography performed during hospitalization. Obstructive coronary disease documented by coronary angiography. Echocardiography post-MI showing EF≥50%. Written informed consent obtained.
Exclusion criteria	Cardiogenic shock or hemodynamic instability	LVEF < 40%; prior cardioverter defibrillator implantation	Allergy/intolerance to beta-blockers	Any condition that may influence the patient’s
	Contraindications to beta-blocker therapy (severe asthma, advanced AV block, bradycardia, hypotension); previous indication for long-term beta-blocker therapy (e.g., heart failure with reduced EF); recent use of beta-blockers for other conditions; pregnant or of childbearing age w/o safe contraception	Contraindications to beta-blocker therapy (hemodynamic instability, bradyarrhythmias, symptomatic HF, severe bronchial asthma and/or COPD)	Absolute contraindication to beta-blocker therapy according to treating physician; history of HF - Killip class on admission or during hospitalization ≥ II; severe valvular heart disease; any condition that requires beta-blocker prescription according to the treating physician; significant risks to patients; patients in other clinical trials	Ability to comply with study protocol; contraindications for beta-blocker therapy; indication for beta-blockade other than as secondary prevention
Outcomes reported	Composite of death from: any cause, MI, unplanned revascularization, ischemic stroke, HF, malignant ventricular arrhythmias (+secondary any of the above individually or pacemaker for 2nd/3rd degree AV block)	Composite of death from: any cause, MI, hospitalization for ACS, hospitalization for HF (+secondary any of the above individually, cardiac death, non-cardiac death, stroke, vasospastic angina, stent thrombosis, major bleeding, target lesion revascularization, any coronary revascularization)	Composite of death from: any cause, reinfarction, hospitalization from HF (+secondary to any of the above individually, sustained vtach, vfib, death from cardiac causes, resuscitated cardiac arrest)	Composite of death from: any cause or new MI (+secondary to either of the above, death from cardiovascular causes, MI, hospitalization for afib, hospitalization for HF)
DOI	10.1056/NEJMoa2505985	10.1371/journal.pone.0199 347	10.1056/NEJMoa2504735	10.1056/NEJMoa2401479

**Table 4:** Certainty of evidence using the GRADE framework GRADE: Grading of Recommendations Assessment, Development and Evaluation; MACE, major adverse cardiovascular events; MI, myocardial infarction

Outcome	No. of Studies	Risk of Bias	Indirectness	Publication Bias	Preliminary Rating	Imprecision	Certainty of Evidence
MACE	4	Serious	Not serious	Not suspected	High	Not serious	Moderate
All-cause mortality	4	Not serious	Not serious	Not suspected	High	Not serious	High
Cardiac death	3	Not serious	Not serious	Not suspected	High	Serious	Moderate
MI	4	Not serious	Not serious	Not suspected	High	Not serious	High
Revascularization	3	Serious	Not serious	Not suspected	High	Not serious	Moderate
Heart failure events	4	Not serious	Not serious	Not suspected	High	Serious	Moderate
Stroke	4	Not serious	Not serious	Not suspected	High	Serious	Low
Malignant ventricular arrhythmia	2	Not serious	Not serious	Not suspected	High	Serious	Low

**Figure 2:**
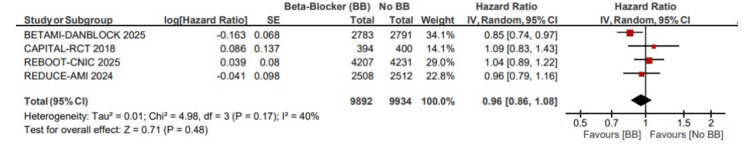
Major adverse cardiovascular events (MACE) Sources: BETAMI-DANBLOCK [17], CAPITAL-RCT [21], REBOOT-CNIC [16], REDUCE-AMI [15]

Across the 4 RCTs including 19,826 patients, MACE occurred in 1,017 of 9,892 patients (10.3%) receiving beta-blocker therapy and in 1,080 of 9,934 patients (10.9%) assigned to no beta-blocker therapy (absolute risk reduction (ARR) 0.6%; number needed to treat (NNT) ≈167). Pooled analysis showed no significant difference between groups (RR 0.95, 95% CI 0.87-1.03; p=0.20). Time-to-event analysis yielded a pooled HR of 0.96 (95% CI 0.86-1.08; p=0.48; I²=40%), consistent in both direction and magnitude. There was low between-trial heterogeneity (8%) (Figure 2).

**Figure 3:**
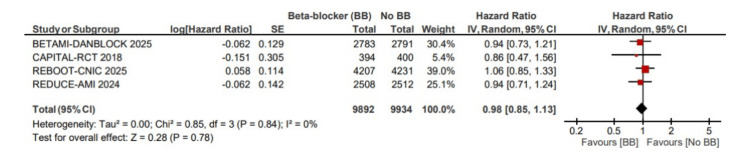
All-cause mortality Sources: BETAMI-DANBLOCK [17], CAPITAL-RCT [21], REBOOT-CNIC [16], REDUCE-AMI [15]

**Figure 4:**
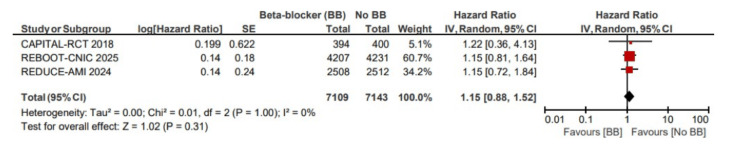
Cardiac death Sources: CAPITAL-RCT [21], REBOOT-CNIC [16], REDUCE-AMI [15]

**Figure 5:**
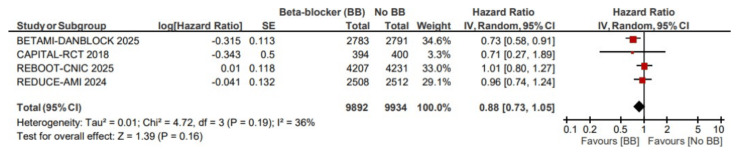
Recurrent MI Sources: BETAMI-DANBLOCK [17], CAPITAL-RCT [21], REBOOT-CNIC [16], REDUCE-AMI [15]

**Figure 6:**
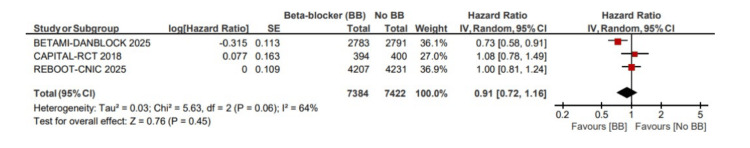
Unplanned Revascularization Sources: BETAMI-DANBLOCK [17], CAPITAL-RCT [21], REBOOT-CNIC [16]

All-cause mortality occurred in 396 of 9,892 patients (4.0%) receiving beta-blockers and 404 of 9,934 patients (4.1%) receiving no beta-blockers (ARR 0.1%; NNT ≈1,000; RR 0.98, 95% CI 0.85-1.13; p=0.84) (Figure 3). No difference was observed in cardiac death (1.5% vs 1.3%; RR 1.15, 95% CI 0.88-1.52; p=0.31) (Figure 4). Recurrent MI occurred in 4.0% vs 4.6% (ARR 0.6%; RR 0.88, 95% CI 0.73-1.05; p=0.16) (Figure 5), and unplanned revascularization in 4.8% vs 4.8% (RR 0.91, 95% CI 0.72-1.16; p=0.45) (Figure 6). HF events were rare (1.1% vs 1.3%; ARR 0.2%; NNT ≈500; RR 0.83, 95% CI 0.64-1.08; p=0.16). Malignant ventricular arrhythmia (0.3% vs 0.4%; RR 0.84, 95% CI 0.47-1.50; p=0.56) and stroke (1.4% vs 1.2%; RR 1.18, 95% CI 0.85-1.64; p=0.31) were infrequent and not significantly different between groups. Heterogeneity remained low across all outcomes. 

In subgroup analyses, beta-blocker therapy was associated with reduced MACE in patients with LVEF 40-49% (RR 0.77, 95% CI 0.62-0.96; p=0.02; I²=0%), whereas no significant benefit was observed in those with LVEF ≥50% (RR 0.97, 95% CI 0.86-1.10; p=0.65; I²=43%). However, this subgroup finding should be interpreted with caution, as the interaction p-value did not reach statistical significance (p=0.07) and the analysis was likely underpowered. Accordingly, this result should be considered exploratory and hypothesis-generating. No significant differences were observed across other subgroups.

Findings were consistent across prespecified sensitivity analyses. Results were unchanged when hazard ratios were pooled, when fixed-effects and random-effects models were interconverted, and when the Hartung-Knapp-Sidik-Jonkman method with restricted maximum-likelihood estimation was applied for between-study variance. None of the outcomes demonstrated sufficient heterogeneity to warrant leave-one-out analyses.

For all-cause mortality, unplanned revascularization, and malignant ventricular arrhythmia, the cumulative evidence crossed the futility boundaries before reaching the DARIS, suggesting that, under the prespecified assumptions of a 10% relative risk reduction, α of 5%, and 80% power, additional trials are unlikely to demonstrate a clinically meaningful benefit for these outcomes. In contrast, all other endpoints, including MACE, recurrent MI, stroke, and HF events, remained inconclusive, with cumulative Z-curves not crossing either the boundary of significance (benefit or harm) or futility boundaries (lack of significance). In the LVEF 40-49% subgroup analysis of MACE, the cumulative Z-curve crossed the conventional significance boundary but did not cross the more stringent O’Brien-Fleming boundary for benefit or the futility threshold. These findings suggest a possible treatment effect in patients with mildly reduced EF. However, the evidence remains insufficient to establish a definitive conclusion.

This meta-analysis of randomized trials evaluating beta-blocker therapy in patients with LVEF ≥40% after AMI yielded 3 principal findings. First, beta-blocker therapy was not associated with a statistically significant reduction in the primary composite outcome of MACE, nor in secondary outcomes, including all-cause mortality, cardiac death, recurrent MI, revascularization, HF events, stroke, or malignant ventricular arrhythmia. Second, while subgroup analyses suggested a potential reduction in MACE among patients with mildly reduced EF (40-49%), this finding should be interpreted with caution. Third, TSA indicated that, under the prespecified assumptions, evidence for selected outcomes such as all-cause mortality and unplanned revascularization has reached futility for a 10% relative risk reduction, whereas other endpoints remain inconclusive.

Our finding of no difference in MACE contrasts with the robust benefits seen in the pre-reperfusion era, when beta-blockers substantially reduced reinfarction and sudden cardiac death [2-4]. Advances in contemporary management, including early revascularization, dual antiplatelet therapy, high-intensity statins, and renin-angiotensin system inhibitors, have substantially reduced baseline ischemic and arrhythmic risk, thereby diminishing the incremental benefit of additional therapies [14,36]. Observational registry data, including the Korea Acute Myocardial Infarction Registry-National Institutes of Health (KAMIR-NIH) study, similarly found no incremental benefit of beta-blockers in patients without HF or reduced EF [37]. 

In contrast, the recently reported BETAMI-DANBLOCK collaboration found that beta-blockers were associated with a significant reduction in MACE, largely driven by fewer recurrent ischemic events [17]. This benefit has been hypothesized to result from negative chronotropic and inotropic effects that prolong diastolic perfusion and reduce myocardial oxygen demand. On the other hand, the REBOOT-CNIC trial, despite enrolling a larger sample, demonstrated no benefit [16]. We propose that several mechanistic factors may account for this discrepancy. BETAMI-DANBLOCK enrolled a greater proportion of patients with mildly reduced EF, the subgroup in which our analysis suggested a potential benefit, and observed higher event rates, increasing the likelihood of detecting a treatment effect. REBOOT-CNIC, on the other hand, included predominantly preserved EF patients, achieved nearly universal use of high-intensity statins, ACEI/angiotensin receptor blockers (ARB), and DAPT, the agents linked with the evidence shift in the reperfusion era. The use of these agents was 10-30% lower in the BETAMI-DANBLOCK trial, thus leaving greater residual risk, which may have contributed to the observed incremental value of β-blockade. Moreover, revascularization strategy was not systematically reported in BETAMI-DANBLOCK, limiting direct comparability. 

The subgroup analysis according to LVEF suggested a possible benefit in patients with mildly reduced EF (40-49%) but not in those with preserved EF (≥50%). However, this observation should be considered exploratory and hypothesis-generating. The interaction p-value did not reach statistical significance, and TSA did not cross the O’Brien-Fleming monitoring boundary for benefit, indicating that the available evidence remains insufficient to establish a true differential treatment effect. Furthermore, subgroup analyses are inherently limited by reduced statistical power and potential for spurious findings. Accordingly, these results should not be interpreted as definitive evidence supporting selective use of beta-blockers in this subgroup but rather as a signal warranting further investigation. 

This signal is nonetheless consistent with prior retrospective studies suggesting that patients with mildly reduced EF may derive benefit from therapies traditionally reserved for reduced EF [38]. Supporting this, Rosselló et al. pooled data from BETAMI, CAPITAL-RCT, DANBLOCK, and REBOOT-CNIC and demonstrated a 25% reduction in a composite of death, MI, or HF among patients with LVEF 40-49% [39]. That analysis, which incorporated harmonized endpoints and time-to-event modeling, offered greater statistical precision and reinforces the hypothesis that beta-blockers may retain clinical value in this population, although the authors acknowledge that this subgroup represented a minority of the overall study population.

No survival benefit was observed with beta-blocker therapy in our pooled analysis (4.0% vs 4.1%; pooled RR ≈0.98), consistent with REDUCE-AMI and REBOOT-CNIC. Contemporary registry data support this conclusion. In a nationwide study, Dondo et al. found no association between beta-blocker use at discharge and lower 1-year mortality in patients without HF or LV dysfunction [40]. In contrast, an earlier observational meta-analysis suggested survival benefit, though largely in settings with reduced EF or limited uptake of secondary prevention [41]. Bangalore et al. further demonstrated that mortality reductions were confined to pre-reperfusion era trials, not in reperfusion-era cohorts [14]. Collectively, these findings reinforce that in current practice, routine long-term beta-blocker therapy after AMI does not confer survival advantage in patients with preserved EF.

HF events were rare in this population (~1% over 3.5 years). The absolute risk difference between treatment arms was only 0.2%, yielding an NNT exceeding 500 to prevent one HF event. In a propensity score-adjusted analysis of older outpatients with HF and EF ≥40% from the PINNACLE registry, beta-blocker use was associated with reduced HF hospitalization in patients with mildly reduced EF (40-49%), but they reported a higher risk of HF hospitalization as EF increased, with potential harm in patients with higher EF, particularly >60% [41]. Similarly, a recent meta-analysis restricted to post-MI patients without reduced EF found no preventive effect of beta-blockers on incident HF in cohorts treated in the modern reperfusion era [42]. These findings are also corroborated by a meta-analysis by Bangalore et al., which reported an increased incidence of HF and cardiogenic shock among patients receiving beta-blockers, conceivably due to their negative inotropic effects [14].

Malignant ventricular arrhythmia occurred infrequently, with no significant differences between groups. In pre-reperfusion era trials such as BHAT and ISIS-1, beta-blockers reduced sudden cardiac death and arrhythmia-related outcomes, reflecting the high arrhythmic risk of untreated infarcts and possible suppressive effect of these agents on substrates [3,4]. In contrast, the absolute incidence of malignant arrhythmia is now extremely low, likely due to early PCI, improved infarct size limitation, and prophylactic ICD therapy in high-risk subgroups, leaving little margin for beta-blocker effect in contemporary practice.

Our findings should also be interpreted in light of clinical heterogeneity in endpoint definitions. Although we attempted to harmonize MACE across trials, the included studies used varying composite endpoints, ranging from narrower definitions (e.g., death and MI) to broader composites incorporating stroke, HF, revascularization, and arrhythmias. While all definitions included core cardiovascular outcomes, pooling non-identical composite endpoints introduces clinical heterogeneity that may not be fully captured by statistical measures such as I². This limitation is inherent to meta-analyses of composite cardiovascular outcomes and may influence the interpretation of pooled estimates.

TSA represents a methodological strength of this study; however, its interpretation depends on prespecified assumptions. We selected a 10% relative risk reduction as the threshold for a clinically meaningful effect, consistent with prior cardiovascular trials. Nevertheless, smaller treatment effects may still be clinically relevant in a common condition such as post-MI secondary prevention. Therefore, crossing futility boundaries for a 10% effect does not exclude the possibility of smaller but meaningful benefits.

The external validity of our findings is limited by the characteristics of the included trial populations. Contemporary RCTs largely enrolled clinically stable patients and excluded individuals with significant HF, hemodynamic instability, advanced conduction disease, or other high-risk features. As such, our results are most applicable to stable post-MI patients with preserved or mildly reduced LVEF receiving contemporary guideline-directed medical therapy, and should not be generalized to all patients with AMI.

From a clinical perspective, our findings do not support a clear benefit of routine long-term beta-blocker therapy in this population. However, absence of evidence should not be interpreted as evidence of absence. The confidence intervals for several outcomes remain compatible with modest benefit or harm. Therefore, decisions regarding beta-blocker therapy should be individualized, taking into account patient-specific risk factors, comorbidities, and tolerance.

In addition, relatively limited data are available regarding patient-centered outcomes such as quality of life, medication adherence, and treatment tolerability. Beta-blockers are associated with potential adverse effects including fatigue, bradycardia, and sexual dysfunction, which may influence adherence and overall treatment burden. As the clinical debate increasingly shifts toward balancing marginal benefit against potential harms, these factors warrant greater consideration in future studies.

This study has several limitations. First, as a trial-level meta-analysis, it is subject to the inherent limitations of the included RCTs and lacks access to individual patient data for more granular risk stratification. Second, although restricted to contemporary trials, the number of included studies was modest, limiting power for rare outcomes such as stroke and malignant ventricular arrhythmia. Third, variability in MACE definitions across trials introduces clinical heterogeneity that may not be fully captured by statistical measures, despite low observed I² values. Fourth, subgroup findings, particularly for patients with LVEF 40-49%, should be interpreted cautiously given limited power and lack of significant interaction testing. Fifth, background medical therapy and revascularization strategies were not identical across studies and may have influenced treatment effects. Sixth, the median follow-up duration may not capture very early or late treatment effects. Finally, TSA depends on prespecified assumptions regarding effect size and statistical thresholds; while futility boundaries were crossed for selected outcomes under a 10% RRR assumption, smaller but clinically meaningful benefits cannot be excluded.

## Conclusions

In this meta-analysis of recent randomized trials, initiation of beta-blocker therapy after AMI in patients with LVEF ≥40% was not associated with a reduction in MACE, mortality, or other major cardiovascular outcomes compared with no beta-blocker therapy. Trial sequential analysis suggests that additional studies are unlikely to alter the null findings for all-cause mortality, revascularization, and malignant ventricular arrhythmias.

A signal toward benefit was observed in the subgroup with mildly reduced LVEF (40-49%); however, this finding remains exploratory and hypothesis-generating, as current evidence is insufficient to support a definitive subgroup effect. In the context of contemporary management, including routine reperfusion and comprehensive secondary prevention, these findings suggest that the routine use of beta-blockers in post-myocardial infarction patients with preserved or mildly reduced LVEF may warrant reconsideration. Nevertheless, treatment decisions should remain individualized pending further evidence. Future patient-level pooled analyses and adequately powered randomized trials are needed to clarify whether specific subgroups, particularly those with mildly reduced LVEF, derive clinically meaningful benefit from beta-blocker therapy.
